# Health-Related Quality of Life after Pediatric Severe Sepsis

**DOI:** 10.3390/healthcare6030113

**Published:** 2018-09-11

**Authors:** Prachi Syngal, John S. Giuliano

**Affiliations:** 1 Pediatric Intensive Care Medicine, Yale-New Haven Children’s Hospital, New Haven, CT 06520, USA; prachi.syngal@yale.edu; 2 Department of Pediatrics, Section Critical Care Medicine, Yale University School of Medicine, New Haven, CT 06520, USA

**Keywords:** HRQL, morbidity, social impact, readmissions, septic shock

## Abstract

Background: Pediatric severe sepsis is a public health problem with significant morbidities in those who survive. In this article, we aim to present an overview of the important studies highlighting the limited data available pertaining to long-term outcomes of survivors of pediatric severe sepsis. Materials and Methods: A review of literature available was conducted using PUBMED/Medline on pediatric severe sepsis outcomes. Long-term outcomes and health-related quality of life (HRQL) following severe sepsis was defined as any outcome occurring after discharge from the hospital following an episode of severe sepsis which affected either the survivor or the survivor’s family members. Results: Many children are discharged with worse clinical and functional outcomes, depending on their diagnosis, treatments received, psychological effects, and the impact of their illness on their parents. Additionally, they utilize healthcare services more than their peers and are often readmitted soon after discharge. However, pediatric HRQL studies with worthwhile outcome measures are limited and the current data on pediatric sepsis is mainly retrospective. Conclusions: There is significant and longstanding morbidity seen in children and their families following a severe sepsis illness. Further prospective data are required to study the long-term outcomes of sepsis in the pediatric population.

## 1. Introduction

Pediatric severe sepsis remains a burdensome public health problem. More than 42,000 children develop severe sepsis each year in the United States alone, and 4400 of these children die [1]. In 1995, Watson et al. reported an incidence of 0.56 cases per 1000 children per year in children under 19 years of age in the US, with the highest mortality noted among neonates [2]. Hartmen et al. showed a steady increase by 81% in the prevalence of sepsis from 1995 to 2005. However, they noted a decline in case fatalities from 10.3 to 8.9% [3]. Unfortunately, these studies may have underestimated the true mortality rate in children with severe sepsis, as they were mostly retrospective studies based on diagnosis codes. Knowing this, an international multicentered prospective point prevalence study was conducted which showed a severe sepsis point prevalence of 8.2% and an increased mortality rate of 25% [1]. Justifiably, most pediatric studies focus on interventions to reduce mortality from sepsis, but emerging data suggest that late outcomes after severe sepsis survival are poor. Morbidity in children following severe sepsis is now similar to that in critically ill adults [1]. In a large prospective trial from European Childhood Life-threatening Disease Study (EUCLIDS) conducted in 52 pediatric intensive care units (PICUs) over seven European countries from July 2012 to January 2016, the authors reported an overall disability rate of 31%, among which 24% was reported in previously healthy children on discharge [4]. Disability was defined as a Pediatric Overall Performance Category (POPC) scale >1, need for skin graft, amputation, or hearing loss. Two other studies showed similar data with disability rates of 28–34% [1,5]. However, recovery remains a long process, with many still feeling effects on their physical, social, emotional, and school functioning for months to years after discharge [6]. Taken collectively, many of these domains form the framework for health-related quality of life (HRQL), simply defined as child’s or parent’s perceived physical and mental health over time. This narrative review focuses on the long-term outcomes following discharge after severe sepsis in children.

## 2. Materials and Methods

This paper consists of a narrative review highlighting key literature available on pediatric severe sepsis outcomes. Studies were identified using electronic databases. Searches were performed in April and May 2018 on PUBMED/Medline. Retrieval of the data was limited to ages 0 to 18 years and to publications in English. Adult literature was included when data on certain topics were absent in children. Index terminology was used, employing Medical Subject Heading (MeSH) along with appropriate keywords. Keywords used for the literature search included: pediatrics, severe sepsis, health-related quality of life (HRQL), pediatric intensive care unit (PICU), morbidity, long-term outcomes, disability, mortality, epidemiology of sepsis, social impact, readmissions, and septic shock. The keywords were generated via synonyms and consulting the existing literature. Titles and abstracts were reviewed for relevance with full articles downloaded when appropriate. Our search identified around 50 articles. Articles not meeting the inclusion criteria were excluded during the review process, resulting in 33 eligible articles. Studies included in the review were publications on pediatric critical illness outcomes, studies on pediatric severe sepsis, and articles on pediatric and adult HRQL. Long-term outcomes and HRQL following severe sepsis were defined as any outcome occurring after hospital discharge from the hospital following an episode of severe sepsis which affected either the survivor or the survivor’s family members.

## 3. Results

Many studies investigating pediatric intensive care outcomes have shown that though most survivors had normal functioning, a significant number of children suffered from substantial psychosocial, physical, and neurocognitive deficits [7,8,9,10,11]. As mentioned above, a recent prospective severe sepsis point prevalence study described morbidity outcomes which lend themselves to future outcome research. The Sepsis Prevalence, OUtcomes, and Therapies (SPROUT) study was conducted over 5 days throughout 2013–2014 at 128 sites in 26 countries and described a pediatric severe sepsis point prevalence of 8.2% and mortality of 25% [1]. Of the survivors, 17% exhibited new moderate to severe functional disability compared to their admission baseline functioning, with mild disability defined as any increase in Pediatric Overall Performance Category (POPC) and moderate disability defined as a discharge POPC score ≥3 and an increase of POPC by ≥1 from baseline [1]. Since these scores were calculated at discharge, almost one in six pediatric severe sepsis survivors were discharged with more disabilities than when they were admitted. It is not clear when these patients return to their pre-admission quality of life, if at all. Farris et al. also described similar results in their review of 384 children who survived severe sepsis by examining the data from the RESOLVE trial (REsearching severe Sepsis and Organ dysfunction in children: a gLobal perspectiVE) [5]. The trial was conducted from November 2002 to April 2005 in 104 study sites in 18 countries. Functional outcome was scored based on POPC score, where poor functional outcome or significant decline in functional status was defined as a POPC score ≥3 and an increase from baseline when measured at discharge or 28 days after trial enrollment. They noted that 34% of survivors exhibited a decline in their functional status at 28 days, as their POPC scores deteriorated one point from the pre-illness score. Moreover, 18% of survivors had poor POPC outcome with a significant decline in their functional status [5]. These results are not unique to pediatrics. Studies examining severe sepsis outcomes in older populations were independently associated with substantial and persistent new cognitive impairment and functional disability among survivors [12].

Children who survive critical illness may develop new or worse neuropsychological functioning as well. A prospective case control study in school-aged children with meningoencephalitis, sepsis, and other disorders followed children for 3–6 months following ICU discharge was performed by Als et al. in 2007 to 2010 [13,14]. The teachers of the survivors described declines in academic performance and neuropsychological testing revealed significant deficiencies while attempting memory tasks when compared to healthy controls [14]. Risk factors for worse neuropsychological impairment were: younger aged children, lower socioeconomic class, and the development of seizures during their admission. Another study conducted to explore neuropsychological outcomes in children admitted to the PICU further investigated problems with memory and found that pattern recognition memory, commonly mapped to the temporal lobe, was most affected in the septic group. These findings suggest that there may be organic changes to the pediatric brain following an episode of severe sepsis [15]. Unfortunately, this is only speculation and future testing to is needed determine its validity. As demonstrated by Rees et al. in 2004, post-traumatic stress disorder (PTSD) was also found to be higher by 21% in severe sepsis survivors within 6–12 months after the PICU discharge, as compared to children discharged from the general ward [16].

The concept of quality of life has seen considerable growth in the past decade. Most of the studies employ the World health Organization’s (WHO) definition of health as their conceptual basis. In 1948, WHO defined health as “a state of complete physical, mental, and social well-being; not merely the absence of disease” [17]. Quality of life has been defined as “an individual’s perception of their position in life, in the context of the culture, environment and value systems in which they live, and in relation to their goals, expectations, standards and concerns”. In pediatric literature, researchers have noted the importance of a child’s developmental age and family’s impact on HRQL [6,17]. Multiple pediatric HRQL tools are available and, despite a lack of consensus on which HRQL tool is best suited for pediatric critical care clinical trials, some key determinants of poor HRQL outcomes in children have been identified. Multiple studies have shown that children with a diagnosis of severe sepsis, especially if the central nervous system was involved, had lower HRQL compared to age-matched peers also admitted to a pediatric intensive care unit (PICU) [5,14]. Others have also shown that this HRQL derangement can last for years [18,19]. Buysee et al. studied patients who survived meningococcal septic shock (MSS) between the years 1988 and 2001 to evaluate the association of MSS patients and different long-term outcomes. They reported a significant association with problem behavior, hence long-term low HRQL based on low emotional and behavioral problem scales up to 2 years after discharge from the pediatric intensive care unit. A lower association was noted with adverse physical outcomes. In addition to physical ailments affecting pediatric HRQL, psychological and social/family determinants also were shown to play an important role in a child’s healing process [6]. Asperberro et al. performed a focused review on studies from 1980 to 2015 and identified key determinants in predicting poor HRQL, including reason for PICU admission (sepsis, meningoencephalitis, trauma), antecedent illness (chronic comorbid conditions), treatments received (prolonged cardiopulmonary resuscitation, long-stay patients, invasive technology), psychological outcomes (post-traumatic stress disorder, parent anxiety/depression), and social and environmental characteristics (low socioeconomic status, parental education and functioning) [6]. A higher level of parental and family stress has been shown to slow down a child’s recovery from the illness, thereby negatively affecting the HRQL.

Besides lower neuropsychological and functional outcomes following an admission for severe sepsis, these children also utilize healthcare more than their peers [2,20]. In a retrospective cohort study conducted in Washington from 1990–2004, nearly half (47%) of pediatric patients formerly admitted with severe sepsis were readmitted at least once, with many readmitted multiple times. These readmissions were typically soon after discharge (median 3 months, IQR 2 days to 14 years) and 85% of these readmissions were emergent [12]. A multivariate regression analysis reported that young age (<1 year), hematologic or neurologic organ dysfunction, bloodstream or cardiovascular infections, and several other comorbidities were independently associated with subsequent readmission [12].

A PTSD diagnosis is significantly more common in families with a child previously admitted to the PICU as well. In a retrospective cohort study conducted in a London teaching hospital from 1998 to 2000, Rees et al. showed that PTSD was 20 times higher in parents of children admitted to the PICU compared to parents of children admitted to the general wards [16]. Other studies have found deteriorating physical health in parents and caregivers of pediatric critical illness survivors compared with adult peers [21,22,23,24,25]. Parents were reported to have physical symptoms such as: numbness, malaise, fatigue, headaches, and irritability. Further, stress-related symptoms such as headache, low energy, and anxiety were reported along with deleterious effects on family health behaviors such as sleep and eating patterns [15,26]. Poor parental coping coupled with decreased levels of patient functioning following PICU discharge only worsen the possibility of these patients returning to their pre-illness state.

Moreover, children are dependent on their families for their physical, emotional, and social needs. Parents of critically ill survivors may have psychological sequelae affecting their own HRQL. A reduction in parental physical and psychosocial well-being has consistently predicted problematic psychological adjustment in the child [22]. The aforementioned studies are summarized in Table 1. 

## 4. Discussion

As mentioned above, HRQL in children is influenced by factors such as the ability to keep up with developmentally appropriate activities and participate in peer group activities [6]. Unfortunately, the vast majority of HRQL literature has focused on the adult patient population. Since the pediatric brain shows more plasticity than their adult counterparts, early intervention may allow children to reach their pre-illness levels of functioning sooner [5]. As described earlier, pediatric HRQL may be more complex since the patient’s family well-being is also deeply impacted [27]. In a study conducted over a period of 5 years, Carnevale found that parent-child bonding strengthened soon after admission to the PICU. Unfortunately, this relationship could become detrimental with more profound critical illness, as some parents devoted so much attention to their critically ill child that they ignored all other responsibilities [28]. Many other studies have also reported similar relationships between the severity of illness and negative family social impact [29,30,31]. 

Many pediatric HRQL tools have been developed, but only a few are considered worthwhile outcome measures [6,32,33]. More than 30 generic and 60 disease-specific HRQL tools have been developed for the pediatric population in the last 20 years [34]. Commonly used tools include the Child Health and Illness Profile (CHIP), the KIDSCREEN-52, the KINDL, and the Pediatric Quality of Life Inventory (PedsQL). Even though these instruments were constructed based on the notion of health, each tool does not measure the same aspect of a child’s health. For example, PedsQL is the shortest of the tools and focuses on physical, emotional, social, and school functioning. KIDSCREEN-52 includes financial resources and autonomy domains. CHIP includes aspects that are related to a child’s future health, such as resiliency and risk avoidance. Importantly, parent proxy report provides unique information and is required when the child is too young to comprehend and report the HRQL tools or unable to act due to physical, psychological, or cognitive problems [34]. Certain factors should be considered when selecting an HRQL measure. Asperberro compiled a list of six factors: psychometric properties of the tool, sensitivity of the tool to changes in time after an intervention, interpretability of the HRQL tool, response burden and other demands placed on the respondent completing the instrument, mode of administration (whether self-report or proxy version), and adaptability of the instrument to be translated to a population different from the original population for which the tool was the first devised [6]. In addition, an adequate pediatric HRQL tool should be able to evaluate a child’s ability to return to their baseline physical, emotional, cognitive, and social health while considering influences from their family and environment [27]. Additionally, it should be able to take into consideration the developmental changes a child experiences over time [17].

As described, prospective data on pediatric long-term outcomes, specifically following severe sepsis, are limited. Many of the epidemiological studies on pediatric sepsis previously discussed are based on retrospective case identification and have reported limited data about long-term, out-of-hospital outcomes [1]. It should be noted that these study designs may be susceptible to recall and selection bias. The few studies that have attempted to prospectively examine long-term outcomes have been largely observational. To date, there have not been any studies focusing on management strategies to improve long-term outcomes in critically ill children with severe sepsis. This area of research is greatly needed. As a result, there are currently no specific guidelines for the follow-up or management of pediatric severe sepsis survivors.

## 5. Conclusions

In conclusion, the short-term outcomes in children following an admission for severe sepsis appear to be poor. Multiple studies have provided data on pediatric long-term outcomes, including HRQL, but assessment tools vary, making meaningful comparisons difficult. Prospective, long-term outcome studies, which may include quality improvement initiatives, are needed to determine modifiable risk factors as mortality from severe sepsis improves but morbidity worsens.

## Figures and Tables

**Table 1:** Long-term outcomes after pediatric severe sepsis.

Long-Term Outcome	Study	Measurement Tool	Result
Functional outcome	Weiss 2015 [1] Farris 2013 [5]	POPC score ≥ 3 and increase in score at 28 days after trial	28–34% of patients had worse POPC scores at discharge
Neuropsychological function	Als 2013 [14] Rees 2004 [16]	Cambridge Neuropsychological Test Automated Battery, the Children’s Memory Scale, the Abbreviated Scale of Intelligence or Wilde Range Intelligence Test PTSD Scale for Children (CAPS-C), the Impact of Event Scale, Strengths and Difficulties Questionnaire, Birleson Depression Scale, Revised Children’s Manifest Anxiety Scale, Child Somatization Inventory	Decreased neuropsychological function 3–6 months following hospital discharge; 21% of children with symptoms of PTSD
Healthcare cost	Watson 2003 [2] Czaja 2009 [12]	Mean length of stay, mean cost, readmissions	Increase of 1.3 million hospital days and $1.97 billion; 50% with additional hospital admission
Impact on family	Board 2004 [15] Rees 2004 [16] Drotar 1997 [22] Klassen 2007 [24] Pochard 2001 [25] Noyes 1999 [26]	Parental Stressor Scale, General Health Questionnaire, Beck Depression Inventory, Hospital Anxiety and Depression Scale	27% higher rate of PTSD, worsening physical health, mental health, and negative social interactions; prevalence of anxiety and depression was 69.1% and 35.4%, respectively

Long-term outcomes after pediatric severe sepsis. PTSD: post-traumatic stress disorder; POPC: Pediatric Overall Performance Category.
